# Deconstructing the DGAT1 Enzyme: Membrane Interactions at Substrate Binding Sites

**DOI:** 10.1371/journal.pone.0118407

**Published:** 2015-02-26

**Authors:** Jose L. S. Lopes, Leila M. Beltramini, Bonnie A. Wallace, Ana P. U. Araujo

**Affiliations:** 1 Instituto de Física de São Carlos, USP, São Carlos, Brazil; 2 Institute of Structural and Molecular Biology, Birkbeck College, University of London, London, United Kingdom; College of Medicine, University of South Florida, UNITED STATES

## Abstract

Diacylglycerol acyltransferase 1 (DGAT1) is a key enzyme in the triacylglyceride synthesis pathway. Bovine DGAT1 is an endoplasmic reticulum membrane-bound protein associated with the regulation of fat content in milk and meat. The aim of this study was to evaluate the interaction of DGAT1 peptides corresponding to putative substrate binding sites with different types of model membranes. Whilst these peptides are predicted to be located in an extramembranous loop of the membrane-bound protein, their hydrophobic substrates are membrane-bound molecules. In this study, peptides corresponding to the binding sites of the two substrates involved in the reaction were examined in the presence of model membranes in order to probe potential interactions between them that might influence the subsequent binding of the substrates. Whilst the conformation of one of the peptides changed upon binding several types of micelles regardless of their surface charge, suggesting binding to hydrophobic domains, the other peptide bound strongly to negatively-charged model membranes. This binding was accompanied by a change in conformation, and produced leakage of the liposome-entrapped dye calcein. The different hydrophobic and electrostatic interactions observed suggest the peptides may be involved in the interactions of the enzyme with membrane surfaces, facilitating access of the catalytic histidine to the triacylglycerol substrates.

## Introduction

Diacylglycerol acyltransferase (DGAT) is an enzyme that catalyses the esterification of a 1,2-diacylglycerol with a fatty acyl-CoA [1], resulting in a triacylglycerol (TAG) molecule in a reaction that is known to be the main limiting step in the TAG synthesis pathway [2].

Homologous DGAT1 genes have been identified in a wide range of eukaryotic organisms, including yeast, plants, fungi, invertebrates and mammals. In tobacco, DGAT1 gene silencing reduced by ~50% the oil content of mature seeds [3]. In *Drosophila*, the DGAT1 gene was identified as causing premature apoptosis [4]. In humans, in addition to the clinical interest in the DGAT1 enzyme as a target for obesity treatment [5–7], studies have found related roles for this enzyme in many human disorders, such diabetes [8], nonalcoholic steatohepatitis [9] and insulin resistance [10]. In cattle, the DGAT1 is associated with the level of TAG deposited intramuscularly and with the regulation of the milk fat content and TAG composition [11, 12].

Little is known about the three-dimensional structure of DGAT1 enzymes as no crystal structures of these or any closely homologous proteins have yet been determined. DGAT1 appears to include multiple transmembrane segments [2]. Similarities with the acylcholesterol acyl-transferase (ACAT) enzymes have been used to predict the potential substrate binding sites of DGAT1 [13]. Studies with the plant *Brassica napus* suggest that a hydrophilic region in the enzyme N-terminus interacts with acyl-CoA [14]. Meanwhile, other studies with DGAT1 from mice suggested that the N-terminal segment is able to specifically bind to different acyl-CoAs [15]. Bovine DGAT1 shares a conserved region with the ACAT enzymes [16], which also suggests a potential binding region for this substrate. Both these binding sites are proposed to lie within an extramembranous loop of the protein.

Substrate binding sites in bovine DGAT1 enzyme that are identical to portions of the ACAT and protein kinase C forms enzymes have been identified [17]. The diacylglycerol (DAG) binding site is located in a soluble extramembranenous loop of the protein, rather than in a transmembrane domain, suggesting it would interact with DAG headgroup on (or close to) the membrane surface [17, 18]. Synthetic peptides (Sit1 and Sit2) corresponding to these proposed substrate binding sites of bovine DGAT1 [17] were shown to interact with the substrates for triacylglycerol synthesis (DAGs and acyl-CoAs) which are present in the membrane. Consequently, it is proposed that the peptides would need to interact with the membrane, in order to access and present the substrates to the catalytic histidine [19] which is located in the same extramembranous loop. In this study, the Sit1 and Sit2 peptides, and a combined version of them into a single peptide, were examined in the presence of model membranes in order to probe whether an interaction between them could enable the binding of the substrates. The knowledge of the DGAT1 binding site structure and the determination of the factors that drive its access to the substrates, together with the identification of the main regions that are required for the enzyme activity will contribute to our understanding of its mechanism of action.

## Material and Methods

### Peptide synthesis, purification and characterization

Synthetic peptides (Sit1, Sit2 and Sit1&2), designed based on the primary structure of the bovine DGAT1 (UniProt code Q8MK44), were obtained as described in Lopes et al [17].

### Liposome preparation

The lipid composition of endoplasmic reticulum (ER) membranes consists of ~50% phosphatidyl choline (PC), 30% phosphatidyl ethanolamine (PE) and 15% (phosphatidyl serine (PS) and phosphatidyl inositol (PI)) [20,21]. These glycerophospholipids vary widely in both the length and saturation of their acyl chains. Different phospholipids were employed here as a simplified system to probe the interaction of the peptides. Large unilamellar vesicles (LUVs) of 1-palmitoyl-2-oleoyl-sn-glycerol-3-phosphate (POPA); 1-palmitoyl-2-oleoyl-sn-glycerol-3-phosphaethanolamine (POPE); 1-palmitoyl-2-oleoyl-glycero-3-phospho-rac-glycerol (POPG); 1-palmitoyl-2-oleoyl-sn-glycero-3-phosphocholine (POPC); 1,2-dipalmitoyl-sn-glycero-3-[phospho-L-serine] (DPPS); 1,2-dipalmitoyl-sn-glycero-3-[phospho-rac-(1-glycerol)] (DPPG); 1,2-dipalmitoyl-sn-glycero-3-phosphocholine (DPPC) were prepared by solubilizing the phospholipids in a mixture of chloroform/methanol 4:1 (v/v); the solvent was then slowly evaporated under a N_2_ stream, yielding a dry lipid film that was subsequently submitted to a SpeedVac system for 2 h. The dry lipids were hydrated with water (or the appropriate buffer), then vortexed and extruded through a polycarbonate filter to yield LUVs with an average diameter of 100 nm (determined by dynamic light scattering). All phospholipids were purchased from Avanti Polar Lipids.

### Synchrotron radiation circular dichroism (SRCD) and circular dichroism (CD) spectroscopy

The SRCD spectra of the DGAT1 peptides (3 mM) were obtained on either the CD1 beamline at ISA synchrotron (Aarhus University, Denmark) or the CD12@ANKA beamline at the ANKA synchrotron (Karlsruhe, Germany). Spectra were collected as an average of 3 scans over the wavelength range from 280 to 170 nm with 1 nm step size and 2s dwell time, at 25° C, using demountable quartz Suprasil cells (Hellma Analytics, UK) with a 0.0009 cm pathlength for both samples and their cognate baselines. CDTool [22] software was used to process the SRCD data, including the averaging of the scans, subtraction of the baseline, smoothing with a Savitsky-Golay filter and calibration versus camphorsulfonic acid. The final spectra were expressed in delta epsilon units.

Conventional CD spectra measurements of the DGAT peptides (50 μM) were recorded from 185 to 280 nm on a J-815 spectropolarimeter (Jasco Instruments, Tokyo, Japan) using a 0.1 cm pathlength rectangular quartz cuvette, as an average of 6 scans. The CD spectra were obtained in 20 mM glycine-HCl (pH 3.0); 20 mM sodium phosphate (pH 7.0) and 20 mM sodium phosphate (pH 11.0) buffers, in the presence and absence of phospholipid vesicles at 50:1 lipid/peptide molar ratios, or in the presence of detergent—either 10 mM sodium dodecyl sulfate (SDS), 1 mM N-hexadecyl-N, N-dimethyl-3-ammonio-1-propanesulfonate (HPS) or 1 mM cetyltrimethylammonium bromide (CTABr).

### Intrinsic fluorescence

Steady state fluorescence emission spectra of the DGAT1 peptides were obtained using an ISS K2 spectrofluorimeter (ISS Fluorescence, Analytical and Biomedical Instruments, Illinois, USA), at 25°C with a circulating water bath using a 1 cm pathlength rectangular quartz cuvette. Samples (50 μM) were excited at 295 nm and the emission spectra recorded from 305 to 450 nm, under the same conditions used in the CD analyses. Reference spectra were recorded and subtracted after each measurement.

### Liposome leakage assays

The release of entrapped calcein in LUVs of POPG was measured to assess integrity of the lipid bilayers in the presence of the peptides. LUVs containing encapsulated probes were prepared as follows: dry POPG was dissolved in a mixture of chloroform/methanol (2:1) and the solvent was slowly removed by evaporation in the presence of N_2_ to form a thin lipid film, followed by lyophilization for 1 h. Lipid films were resuspended in 5 mM HEPES (pH 7.4) containing 35 mM calcein, submitted to a series of 10 freeze/thaws cycles and extruded 11 times using polycarbonate filters (Nucleopore) with 100 nm pore diameters. These vesicles were applied onto a Sephadex *G*-75 column and eluted with 5 mM HEPES (pH 7.4) containing 100 mM NaCl to remove the unencapsulated calcein. The LUV suspension was diluted in 5 mM HEPES (pH 7.4) containing 100 mM NaCl to give a final lipid concentration of 0.1 mM. The final concentration of phospholipid was determined by phosphorus analysis [23]. Different amounts of the peptides were added to the cuvette and the extent of leakage was measured at different peptide:lipid molar ratios. Fluorescence intensities were recorded at 25° C with continuous stirring. Measurements were performed as a function of time with excitation at 490 nm and emission at 520 nm. The percentage of leakage was calculated by using the following equation:

$${\%}_{Leakage}=\frac{\left(F-F_{0}\right)}{\left(F_{100}-F_{0}\right)}\times 100,$$

where *F*_*0*_ is the initial fluorescence intensity of vesicles, *F* is the fluorescence intensity after adding the peptide, and *F*_*100*_ is the fluorescence after addition of Triton X-100 (to completely solubilize and disrupt the LUVs).

### Surface plasmon resonance

Biosensor experiments were carried out using a BIAcore X analytical system (GE Healthcare) with an L1 sensorchip. The immobilization of the phospholipids was performed as follows: Firstly, N-octyl-LD-glucopyranoside (40 mM) was used to clean the sensorchip surface. Then, DPPG LUVs (1 mM, diameter 100 nm) were injected onto the sensorchip surface for 50 min, with a 1 μl/min flow, at 25°C. After this time, the flow was increased to 100 μl/min for 10 min, and 4 mM NaOH was injected into the cell for 1 min, with a 5 μl/min flow. Finally, bovine serum albumin (1.5 μM) was injected in order to eliminate nonspecific interactions. Peptides (from 0.15 μM to 100 μM) were then injected and their interactions with the bilayer were monitored for 10 min, with a 20 μl/min flow rate. The sensorgrams were analyzed with the BIAevaluation software Version 4.1. The running buffer was 10 mM HEPES (pH 7.4) with NaCl (150 mM) and EDTA (1mM), and the regeneration solution was NaOH (10 mM). All solutions were freshly prepared and degassed prior to use.

## Results and Discussion

### Conformations of DGAT1 peptides in solution

**Fig 1:**
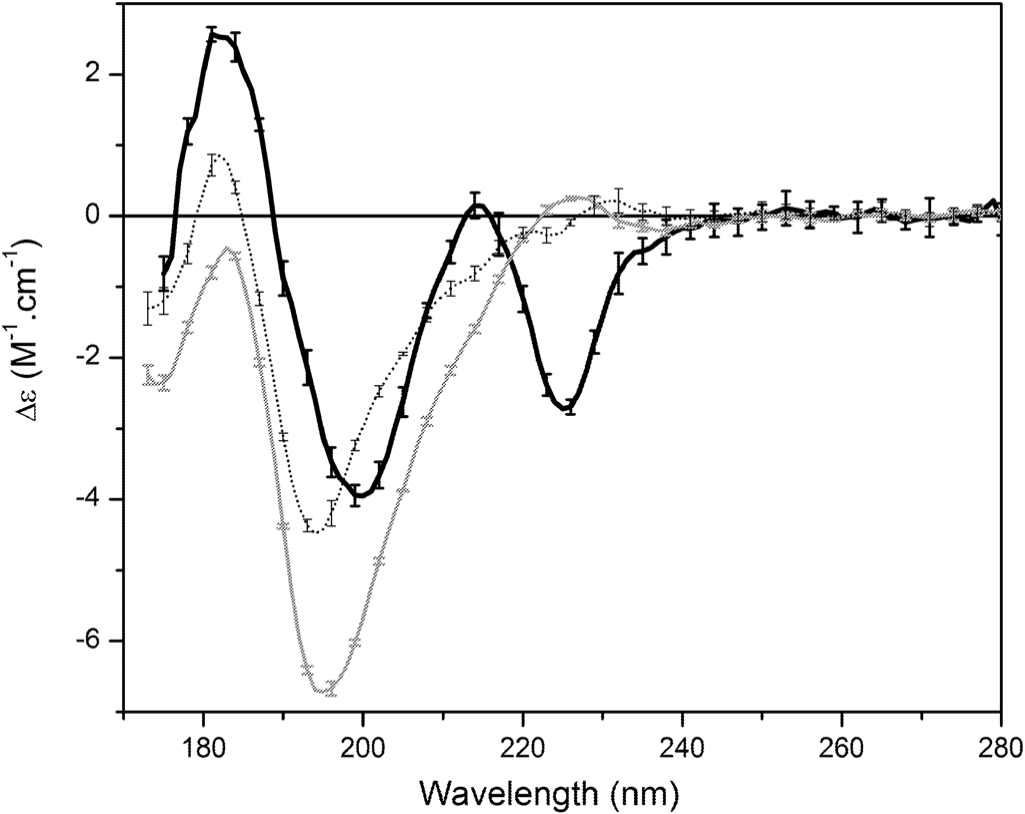
SRCD spectra of DGAT1 peptides. Sit1 (black), Sit2 (grey) and Sit1&2 (dot) in 20 mM Sodium Phosphate buffer (pH 7.0). Error bars represent one standard deviation in the replicate measurements.

The SRCD spectra of the peptides in aqueous solution, (Fig. 1) were consistent with a predominantly unordered secondary structures for all three isolated peptides in solution, as indicated by their negative peaks at ~200 nm [24, 25] and small positive peaks around 182 nm. In the SRCD spectrum of Sit1, the presence of multiple adjacent aromatic residues Trp appears to give rise to a peak with a minimum at 228 nm (suggestive of exciton coupling of aromatic residues), in a similar way to antimicrobial peptide tritrpticin [26], a 13 residue peptide with adjacent Trp residues (VRRFPWWWPFLRR). The high flux of the SRCD spectroscopy has greatly improved the signal-to-noise ratio in the data, reducing the differences between the replicates. Analysis of the errors bars with the individual scans and the replicates of the CD/SRCD measurements have indicated only small deviations on each sample even at the low wavelength region.

The fluorescence emission spectra of these peptides in aqueous solution confirmed the exposure of the Trp residues to the aqueous environment, since maximum emission was centered at 354 nm, a wavelength corresponding to the Trp emission when free in aqueous solution [27].

Only small differences in the CD spectra were observed when the peptides were incubated at different pHs from 3.0 to 11.0 (S1 Fig.), and likewise, no displacement of the fluorescence maximum emission was observed as a function of pH. However, quenching was noted at the pH 3.0 (S1 Fig.), which could be explained by the protonation of the histidine residues in Sit2 at acid pH, as they could act as strong fluorescence quenchers, with a charge transfer between the indole and the imidazole ring when they approach each other.

### Interactions with model membranes

In order to investigate the interaction of DGAT1 peptides with the model membranes, peptides were incubated with detergent or phospholipid vesicles. Initially, zwitterionic, and negatively- and positively- charged surfactants (SDS, HPS and CTABr, respectively) were used to examine whether the head group net charge could favor interaction with any of the peptides.

**Fig 2:**
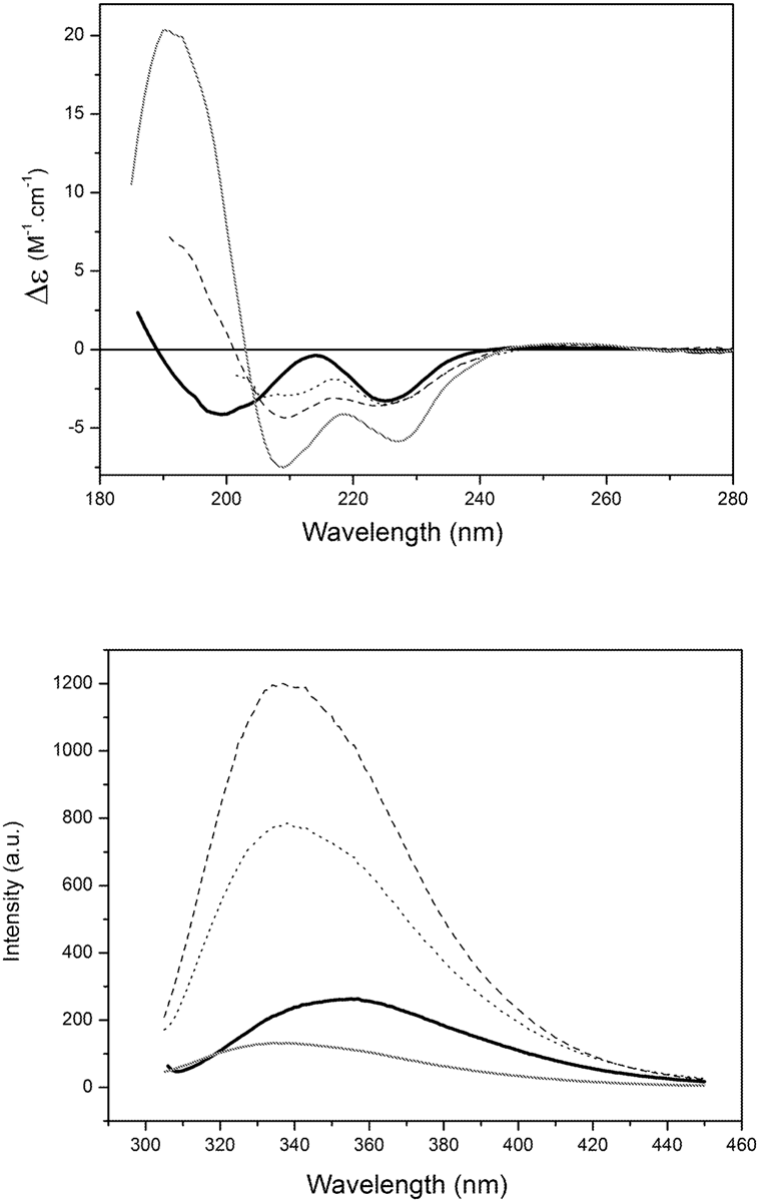
Interactions of the Sit1 peptide with different types of model membranes. a) CD spectroscopy Sit1 in the presence of micelles composed of HPS (dash), CTAB (dot), and SDS (grey), compared with the peptide in aqueous solution (black). b) Emission spectra of Sit1 in aqueous solution (black) and in the presence of HPS (dash), CTAB (dot), and SDS (grey) detergents.

The CD spectrum of the peptide Sit1 exhibited a conformational change, indicated by the presence of new spectral minima at 208 nm (Fig. 2a) in the presence of all the three detergents. In agreement with the CD data, a blue shift was observed in the fluorescence maximum emission of Sit1 in the presence of all the detergents (Fig. 2b), changing from 354 to 337 nm. This suggests the aromatic sidechains of the Trp residues are less exposed to the aqueous environment and perhaps are accessible to the hydrophobic core of the detergent micelles.

The CD spectra of the peptide Sit2 showed a dependence on the nature of the hydrophilic head group component of the detergents, with the most prominent conformational change occurring in the presence of the SDS (S2 Fig.), producing peaks at 206 and 222 nm, and a low intensity positive band at 195 nm. Only very minor spectral changes were observed for Sit2 with the positively-charged surfactant, suggesting a lack of interaction. In the presence of the zwitterionic HPS micelles, a decrease of the intensity of the peak at 198 nm and a small increase of the band at 222 nm, might suggest a slightly more ordered net structure for Sit2 than it was in aqueous solution.

The fluorescence spectra of Sit2 in the presence of the detergents also indicate a clear interaction between the peptide and the negatively-charged detergents, with a blue shift in the λ_max_ to 331 nm suggesting a sequestering of the aromatic residues from interactions with the solvent. A blue shift was also seen when Sit2 was incubated with the HPS micelles, in agreement with the changes observed by CD. The increase in the fluorescence intensity observed for peptides with HPS suggests it interacts differently from the other zwitterionic surfactants due to the higher degree of hydration of its polar head groups [28, 29]. Finally, for Sit2, essentially no binding was detected in the positively-charged micelles (CTAB) by the fluorescence assays, probably due to the repulsion of the peptide with its basic pI from the positive surface of the micelle (S2 Fig.), in agreement with the CD results.

The interaction of Sit1&2 with micelles followed a pattern similar to that observed for Sit2 (S2 Fig.), indicating involvement of charge interactions between peptide and detergents, which result in conformational changes in the peptide structure.

In order to evaluate the interaction of the peptides with lipids, LUVs comprised of phospholipids with different head groups and fatty acid tails were incubated with the DGAT1 peptides. The interaction of Sit1 with the phospholipids presented a different behavior from that observed with the detergents. No significant changes in the CD spectra of Sit1 were observed when it was incubated with either zwitterionic or charged vesicles, producing similar spectra to those found in aqueous solution (S3 Fig.). It seems probable that the access of the peptide Sit1 to the acyl chains region was prevented, since this hydrophobic region in the liposomes is not as easily accessible as in micelles.

**Fig 3:**
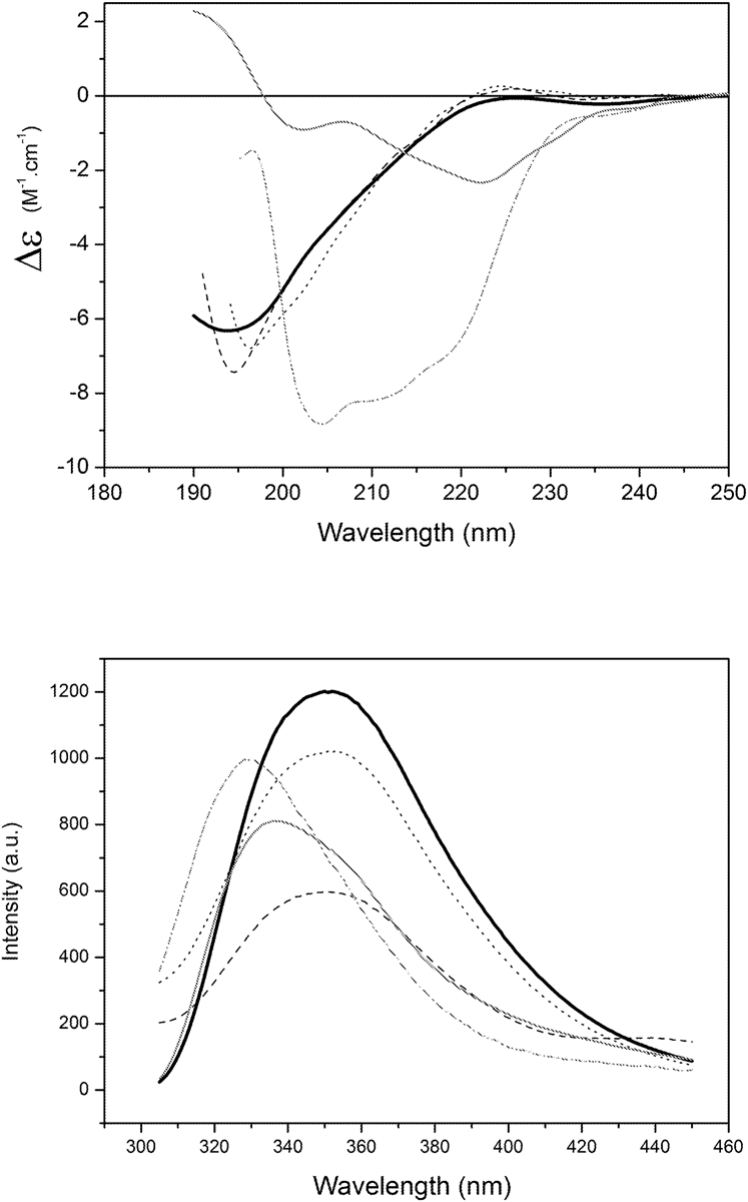
Interactions of the Sit2 peptide with different types of model membranes. a) CD spectra of Sit2 in aqueous solution (black) and in the presence of LUVs composed of different phospholipids (DPPS (dash), DPPC (dot), POPA (grey), and DPPG (dash dot grey)). b) Fluorescence spectra of Sit2 in aqueous solution (black) and in the presence of the different LUVs.

Sit2 also exhibited no alteration of its CD spectrum when incubated with zwitterionic LUVs. However, its CD spectrum changed considerably in the presence of negatively-charged LUVs (PG and PA) (Fig. 3a). Additionally, a blue shift of 15 nm was observed in the λ_max_ of the Sit2 fluorescence spectrum (Fig. 3b), also suggesting the microenvironment of this peptide was altered in the presence of negatively-charged membranes. This interaction may have been induced by the electrostatic attraction with positively-charged residues on the peptide.

The interaction of Sit1&2 with LUVs (S4 Fig.) was very similar to that for Sit2, with little alteration observed for the zwitterionic vesicles, but producing an altered CD spectrum (perhaps indicative of a more ordered net peptide structure) in the presence of the POPG negatively-charged LUVs; this is in agreement with the blue shift of the fluorescence λ_max_, indicating the aromatic residues are more sequestered from interactions with aqueous environment. Surprisingly, in the presence of the POPA LUVs, only a small shift of the peak at 198 nm of the CD spectrum was observed, suggesting only a modest change in the peptide structure. Negatively-charged PA presents a very small headgroup when compared to the other negatively-charged lipids (PGs or PCs), perhaps because this phospholipid produces a negative spontaneous curvature whereas PG produces zero spontaneous curvature [30].

### Disruption of the membranes: Calcein leakage assays

**Fig 4:**
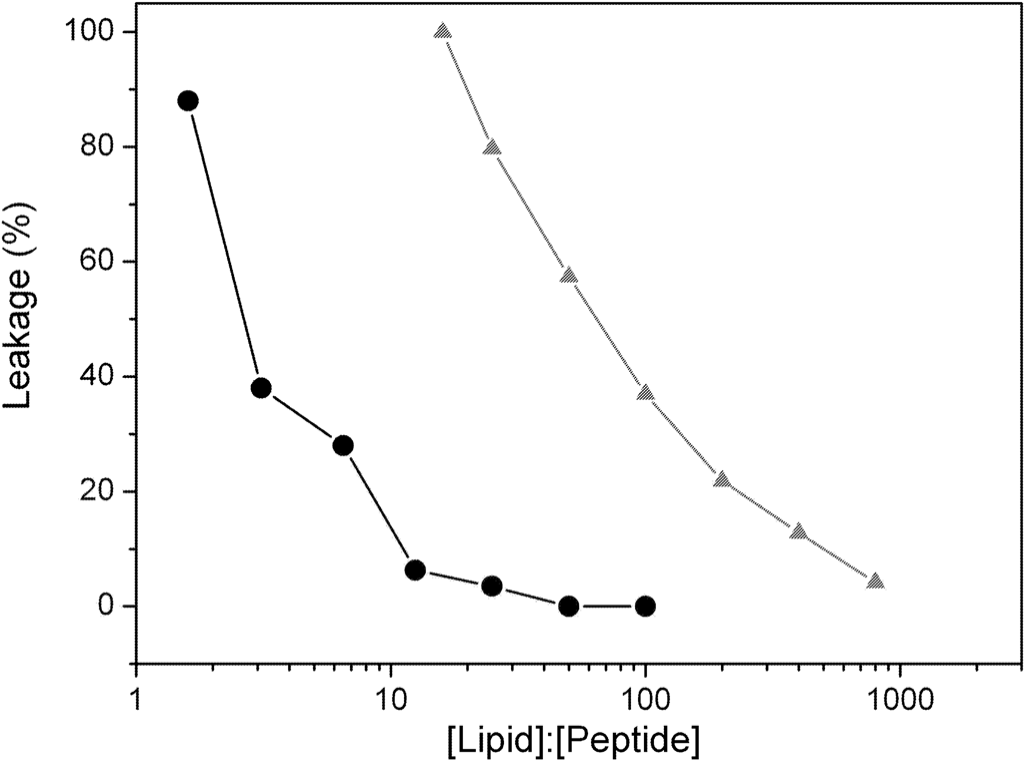
Integrity of LUVs in the presence of the peptides. Comparative leakage curves of Sit1 (black circles) and Sit2 (grey triangles) at different lipid:peptide molar ratios, after 10 min of incubation at 25°C.

The purpose of these assays was to compare the ability of the DGAT1 peptides to disturb the integrity of the lipid bilayers formed by negatively-charged phospholipids. The kinetics of calcein release from POPG vesicles (S5 Fig.) differed for Sit1 and Sit2 peptides. The addition of both Sit1 and Sit2 (Fig. 4) resulted in permeabilization of the liposomes and the release of the probe from the inside; however, different peptide:lipid ratios were needed to promote the complete leakage of the vesicles. As the effects of binding of Sit2 to the POPG liposomes were more pronounced, its disruptive effect on the LUVs integrity also occurred at a lower peptide:lipid (1:100) ratio than the ratio (1:2) needed to produce comparable results for Sit1.

### Binding to membranes: Surface plasmon resonance (SPR)

**Fig 5:**
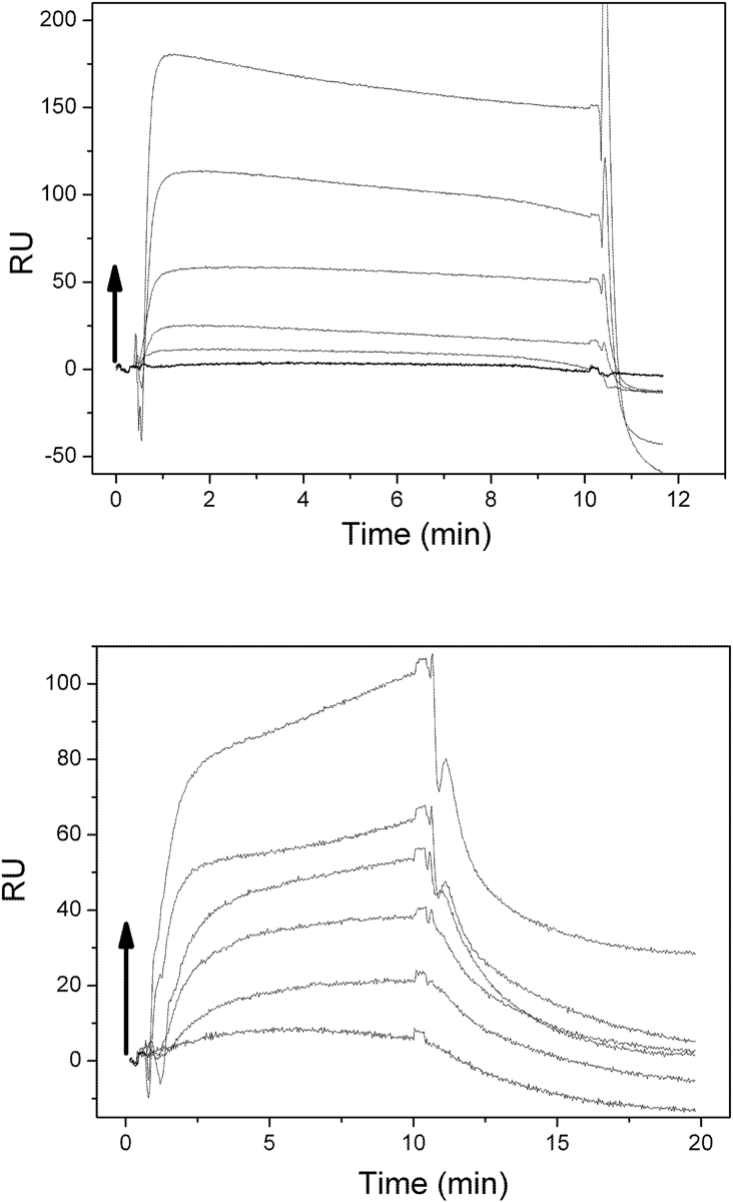
Surface plasmon resonance sensorgrams. a) Sit1 (at 0, 5, 10, 25, 50, and 100 μM) and b) Sit2 (at 0.15, 0.31, 0.62, 1.25, 2.5, and 5 μM) on DPPG bilayers immobilized on a L1 sensorchip. The black arrows indicate the times of additions of peptide and the direction of the increase of peptide concentration.

The interaction of the peptides with lipid bilayers was investigated using SPR to assess the adsorption and dissociation processes. The sensorgrams showing the kinetics of the binding of Sit1 (5 to 100 μM) and Sit2 (from 0.15 to 5.0 μM) to the lipid surface were monitored for 10 min (Fig. 5). The amount of peptide bound on the phospholipid surface was proportional to the concentration of the peptide injected on the sensorchip for both Sit1 and Sit2 peptides. After the initial time, the spontaneous dissociation of the peptides from the lipid surface was also monitored and revealed that the total dissociation of Sit1 occurred in the first moments after the final injection of the peptide onto the lipid surface, confirming its weak interaction with the negatively-charged liposomes, as had been observed using CD and fluorescence spectroscopies. For Sit2, however, the dissociation occurred more slowly, since the peptide was able to be efficiently bound to the negatively charged bilayer. According to the sensorgrams, the dissociation constants (K_d_) from the DPPG vesicles were calculated to be 170 μM and 0.43 μM for Sit1 and Sit2, respectively. The stronger interaction of Sit2 with negatively-charged surfaces suggest that charge interactions can play a role in modulating the binding of this segment of the DGAT1 enzyme to the substrates and/or the biologic membranes. Therefore, the presence of charged domains in biological cell membranes could trigger the attraction of the segments from the external loop of the DGAT1 protein to the membrane surface, where the substrates for triacylglycerol synthesis are localized.

## Conclusions

The use of the model membrane systems allowed us to distinguish between different modes of interaction of the predicted peptides involved in DGAT1 activity. One of the binding sites (Sit1) appears to involve interactions with the lipid acyl chains of fluid model membranes, which allows the access of the peptide to the hydrophobic core of the system, while in the other binding site (Sit2), may involve an electrostatic attraction to negatively-charged membranes head groups. Nevertheless, the interactions of the putative binding sites in the intact DGAT1 enzyme are likely to be more complicated than those of the isolated model peptides examined in this study, since their local environment, net charge, or position within the enzyme could influence their ability to interact with membrane bilayer. This may be especially true for the region represented by the Sit2 peptide, where its net charge may bias its ability to bind to specific types of head groups. However, the information provided by this study regarding peptides representing proposed binding sites of the DGAT1 enzyme in the presence of lipid systems (membranes and substrates) do provide new insights about this important enzyme and its interactions. Since these peptides correspond to the predicted binding sites of the two substrates of DGAT1, knowledge of the factors that govern their ability to undergo conformational changes provide some new clues about how the enzyme’s activity can be modulated.

Given the DGAT1 enzymes represent interesting therapeutic targets in obesity [5–7], insulin resistance [10, 31], and in hepatitis C virus infection [32], it is important that DGAT1 structure-activity relationships are characterized, especially in the absence of any crystal structure of this enzyme or a close homologue. The information provided by this study on synthetic peptide corresponding to substrate binding sites should therefore contribute to understanding of the functioning of theses enzymes in metabolic disorders [33], and the development of novel inhibitors [34].

## Supporting Information

S1 Fig Minimal effect of pH on the structure of the DGAT1 peptides. CD spectra of a) Sit1, b) Sit2 and c) Sit1&2 in pH 3.0 (dash), pH 7.0 (bold) and pH 11 (dot). Fluorescence spectra of d) Sit1, e) Sit2 and f) Sit1&2. Excitation was performed at 295 nm, and emission was monitored from 305 to 450 nm.
(TIF)

S2 Fig Interactions of peptides with detergents. CD spectra of a) Sit2 and b) Sit1&2 in water (black), in the presence of the detergents HPS (dash), CTAB (dot), and SDS (grey). Fluorescence spectra of c) Sit2 and d) Sit1&2 with the same detergents.
(TIF)

S3 Fig Interactions of the Sit1 peptide with LUVs. a) CD spectra of Sit1 in water (black) or with LUVs of DPPS (dash), POPC (dot), POPA (grey), DPPG (dash dot grey), and POPG (dash dot dot grey). b) Fluorescence spectra of Sit1 (bold) in water or with LUVs of DPPS (dash), POPC (dot), POPA (grey), DPPG (dash dot grey), and POPG (dash dot dot grey).
(TIF)

S4 Fig Interactions of the Sit1&2 peptide with LUVs. a) CD spectra of Sit1&2 in water (black) or with LUVs of POPC (dash), POPE (dot), POPA (grey) and POPG (dash dot grey). b) Emission spectra of Sit1&2 in water (black) or with LUVs of POPC (dash), POPE (dot), POPA (grey) and POPG (dash dot grey).
(TIF)

S5 Fig Release of the internal contents from POPG liposomes. The addition of increasing amounts of a) Sit1 (8, 16, 32, 32, and 64 μM) and b) Sit2 (0.125, 0.25, 0.5, 1, 2, 4, and 6 μM) gradually disturbs the liposome integrity. The arrows indicate the additions of peptide, and the bold curve is following addition of Triton-10% detergent (after 10 min incubation), which completely disrupts the bilayer.
(TIF)
