# Correction: Deconstructing the DGAT1 Enzyme: Membrane Interactions at Substrate Binding Sites

**DOI:** 10.1371/journal.pone.0124336

**Published:** 2015-04-09

**Authors:** The PLOS ONE Staff

The third author’s name is spelled incorrectly. The correct name is: B.A. Wallace.
